# Moderate exercise training attenuates aging-induced cardiac inflammation, hypertrophy and fibrosis injuries of rat hearts

**DOI:** 10.18632/oncotarget.6168

**Published:** 2015-10-19

**Authors:** Po-Hsiang Liao, Dennis Jine-Yuan Hsieh, Chia-Hua Kuo, Cecilia-Hsuan Day, Chia-Yao Shen, Chao-Hung Lai, Ray-Jade Chen, V. Vijaya Padma, Wei-Wen Kuo, Chih-Yang Huang

**Affiliations:** ^1^ Graduate Institute of Basic Medical Science, China Medical University, Taichung, Taiwan; ^2^ School of Medical Laboratory and Biotechnology, Chung Shan Medical University, Taichung, Taiwan; ^3^ Laboratory of Exercise Biochemistry, Department of Sports Sciences, University of Taipei, Taipei, Taiwan; ^4^ Department of Nursing, Mei Ho University, Pingguang Road, Pingtung, Taiwan; ^5^ Division of Cardiology, Department of Internal Medicine, Armed Force Taichung General Hospital, Taichung, Taiwan; ^6^ Department of Surgery, School of Medicine, College of Medicine, Taipei Medical University, Taipei, Taiwan; ^7^ Department of Biotechnology, Bharathiar University, Coimbatore, India; ^8^ Department of Biological Science and Technology, China Medical University, Taichung, Taiwan; ^9^ Graduate Institute of Chinese Medical Science, China Medical University, Taichung, Taiwan; ^10^ Department of Health and Nutrition Biotechnology, Asia University, Taichung, Taiwan

**Keywords:** aging, cardiovascular disease, heart failure, moderate exercise training, Gerotarget

## Abstract

Aging is the most important risk factor in cardiovascular disease (CVD), which is the leading causes of death worldwide and the second major cause of death in Taiwan. The major factor in heart failure during aging is heart remodeling, including long-term stress-induced cardiac hypertrophy and fibrosis. Exercise is good for aging heart health, but the impact of exercise training on aging is not defined. This study used 3-, 12- and 18-month-old rats and randomly divided each age group into no exercise training control groups (C3, A12 and A18) and moderate gentle swimming exercise training groups (E3, AE12 and AE18). The protocol of exercise training was swimming five times weekly with gradual increases from the first week from 20 to 60 min for 12 weeks. Analyses of protein from rat heart tissues and sections revealed cardiac inflammation, hypertrophy and fibrosis pathway increases in aged rat groups (A12 and A18), which were improved in exercise training groups (AE12 and AE18). There were no heart injuries in young rat hearts in exercise group E3. These data suggest that moderate swimming exercise training attenuated aging-induced cardiac inflammation, hypertrophy and fibrosis injuries of rat hearts.

## INTRODUCTION

Aging is an inevitable trend of the world's population, and it is accompanied with serious age-related health issues in modern society that must be investigated. The risks for high cholesterol, hypertension, ventricular hypertrophy, diabetes, cerebrovascular disease, ischemic heart disease, myocardial infarction, obstructive sleep apnea (OSA), changes in tissue macrophages and related cancers increase with aging [1-6]. According to World Health Organization statistics, heart disease is the leading causes of death in the world. Moreover, The Department of Health in Taiwan published the top ten leading causes of death and reported that cardiovascular diseases (CVDs) are the second major cause of death [7] and lead to atherosclerosis, hypertension and heart failure.

The process of heart failure includes stress-induced cardiac remodeling, cardiac apoptosis, cardiac fibrosis and heart failure. Cardiac remodeling in CVD is one of the most frequently occurring characteristics of aged hearts [8, 9]. The risk reasons for cardiac remodeling during aging include heredity, diabetes mellitus, obesity, hypertension, hyperlipidemia, and smoking [10]. Previous studies indicated that aging induced an inflammatory response in the rat heart [11, 12]. Tumor necrosis factor α (TNF-α), nuclear factor-κB (NF-κB) and cyclooxygenase 2 (COX-2) are the major protein markers of the cardiac inflammation pathway [13, 14]. Inflammation, myocardial injury and hypertension results from pathological hypertrophy, which cannot be reversed [15]. The interleukin 6 (IL-6) signaling pathway is a major pathway that regulates cardiac hypertrophy, IL-6 and IL-6 receptor-activated downstream genes, such as the mitogen-activated protein kinase 5 (MEK5), extracellular signal-regulated kinase (ERKs) and calcineurin, which promotes the translocation of transcription factor-signal transducer and activator of transcription 3 (STAT3) and GATA binding protein 4 to the nucleus [16]. Previous research on the concentric hypertrophy pathway demonstrated that mitogen-activated protein kinase (MEK1/2) activated ERK1/2 and c-Jun N-terminal kinase (JNK), which induced calcineurin expression and promoted the transcription factor nuclear factor of activated T-cells, cytoplasmic 3 (NFATc3) into the nucleus [17] and translocated downstream gene b-type natraretic peptide (BNP), the levels of BNP which is a risk for heart disease [18]. Some studies indicated that activation of the MEK5/ERK5/STAT3 signaling pathway promoted eccentric hypertrophy, which is one type of pathological hypertrophy [19, 20].

Fibrosis is the end-stage step after cardiac myocyte apoptosis [21]. Fibroblasts result in fibrosis, which leads to reduced cardiac physiological functions [22]. Heart injury and remodeling, such as inflammation and hypertrophy, induce cardiac fibrosis, which plays an important role in progression of heart disease, but the mechanisms and risk factors of fibrosis are not clear [23]. Our recent paper demonstrated that transforming growth factor beta (TGF-β) activated downstream signaling pathways and increased connective tissue growth factor (CTGF) [24]. CTGF expression in the fibrosis pathway upregulated and translocated to the cell nucleus, which leads to fibroblast proliferation [25]. Our previous paper demonstrated that another fibrosis pathway of fibroblast growth factor 2 (FGF-2) activated urokinase/tissue-type plasminogen activator (uPA/tPA) and matrix metalloproteinase 2 (MMP-2) in cardiomyoblast cells [26]. Moderation of exercise training prevents colonic inflammation via regulation of peroxisome proliferator-activated receptor gamma in obese mice [27], and exercise training reduced pathological cardiac hypertrophy, which was induced by pressure overload [28]. Another paper indicated that exercise training reduced vascular fibrosis in obese rats [29]. This research demonstrated that exercise training improved some protective function, but there was no evidence indicating that exercise training prevented heart injury in natural aging rats.

Our previous study discovered that exercise training decreased aging-induced cardiomyocyte apoptosis, which is the end-step in heart failure progression, and long-term stress followed the age increase in cardiac inflammation, hypertrophy and fibrosis. [7]. Therefore, suitable exercise training prevented heart injury by reducing heart failure progression caused by natural aging in rat heart, as expected [30, 31].

Exercise training decreased the risk for heart failure [32] and improved cardiac function [9, 33]. This study used moderate exercise training of open and free swimming in warm water at a suitable depth in a swimming pool to avoid excessive fear of drowning and struggle. The effects of this moderate swimming exercise training on aging-induced cardiac inflammation, hypertrophy and fibrosis injuries in rat hearts were investigated.

## RESULTS

### Exercise training improve inflammation in aging rats

**Figure 1:**
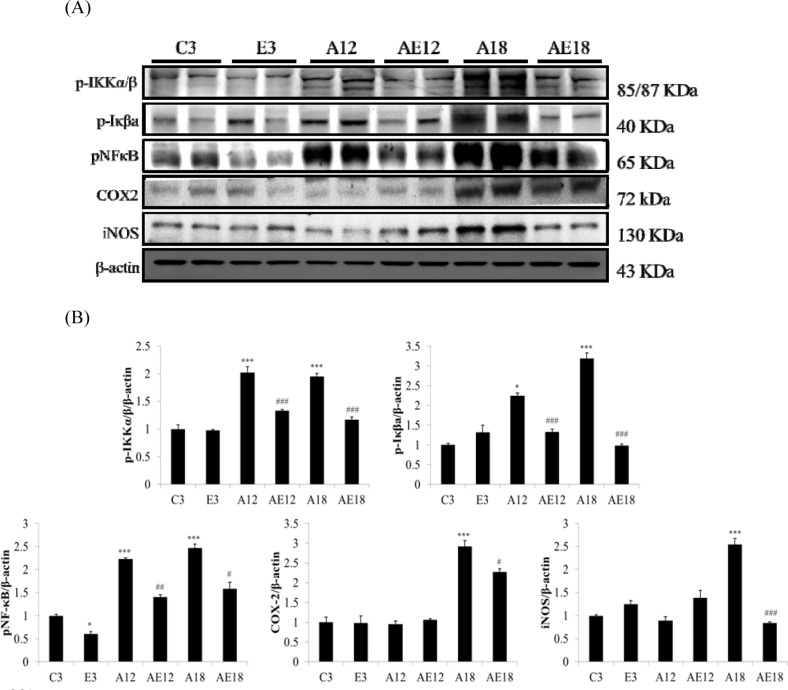
The representative inflammatory protein products extracted from the left ventricles of two rats in each group: control rats (C3), aging rats (A12, A18), and aging, exercise-trained rats (E3, AE12, AE18) were measured using Western blotting β-actin was used as an internal control. *=*P* < 0.05, **=*P* < 0.01, *** =*P* < 0.001 compared to the age control group (C3 group); #=*P* < 0.05, ##=*P* < 0.01, ### =*P* < 0.001 compared to the indicated groups without exercise training.

We measured TNFα, p-IKKα/β, p-Iκβa, pNFκB, COX2 and iNOS to investigate the major pathways of cardiac inflammation in aging rats and aging rats after exercise training (Fig. 1A). The aging groups (A12 and A18) exhibited myocardial inflammatory compared with the younger group (C3). The cardiac inflammation was age-dependent because inflammation in the A18 group was more severe than the A12 group. Cardiac inflammation was down-regulated in the E3, AE12 and AE18 group after exercise training. Notably, exercise training reduced the expression of members of the inflammation signaling pathway in aging groups (AE12 and AE18) better than the young group (E3), especially in the AE18 group.

### Body weight, cardiac histopathological analysis and heart weight change

Rat body weights (BW) were measured and analyzed. Table 1 shows that the BW of rats increased with increasing age, and BW decreased after exercise training, especially in the aging groups (AE12 and AE18). Rat heart tissues were analyzed to compare differences between groups. Residual adipose tissue and any remaining blood was removed, and paper was used to dry excess water. Whole heart (HW) weight and tibia length were determined in each group.

**Table 1:** Characteristics of aging rats with or without exercise training (A and AE)

	C3 (n=5)	E3 (n=5)	A12 (n=5)	AE12 (n=5)	A18 (n=3)	AE18 (n=3)
BW (g)	531.00±43.88	494.86±71.24	701.74±79.51 **	605.64±49.2 *#	769.70±25.14 ***	650.07±29.55*##
HW (g)	1.42±0.07	1.43±0.06	1.69±0.08 ***	1.52±0.06 #	1.86±0.05 ***	1.65±0.09 **#
HW/Tibia (mg/mm)	30.64±1.48	30.79±1.55	34.15±1.47 ***	31.1±1.15 #	37.00±1.10 ***	32.73±1.49 ##

Values are means±SD

BW: body weight

HW: whole heart weight

*= *P* < 0.05, **=*P* < 0.01, *** =*P* < 0.001 compared to the age control group (C3 group)

#=*P* < 0.05, ##=*P* < 0.01, ### =*P* < 0.001 compared to the indicated groups without exercise training.

**Figure 2:**
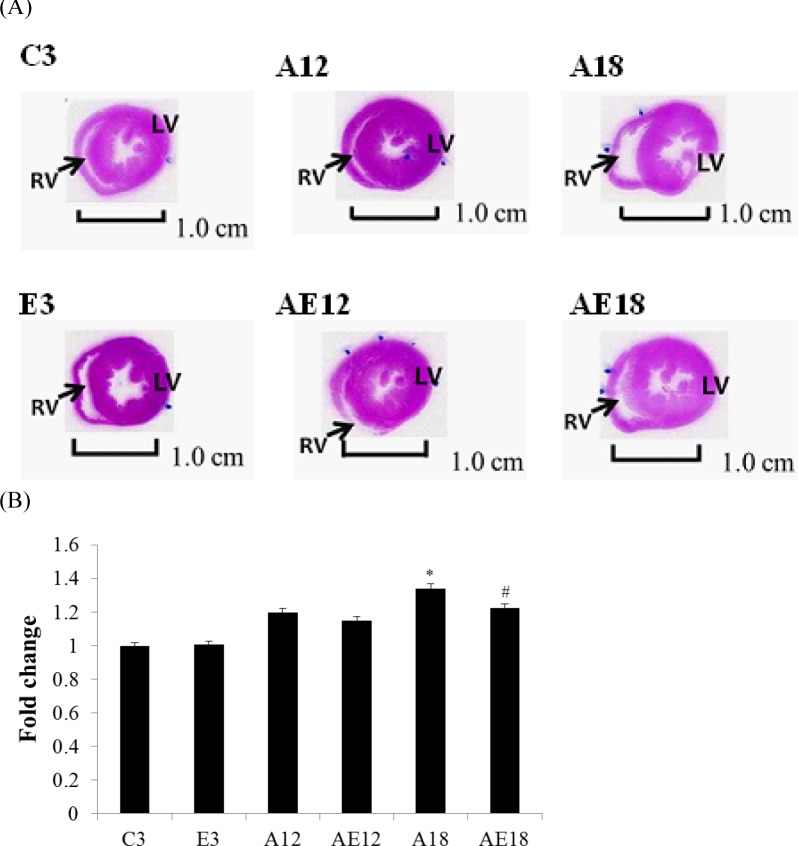
Hematoxylin and eosin staining Cardiac tissue sections stained with hematoxylin and eosin. (A) The myocardial architecture images were magnified ×10. The scale bar is 1 cm. RV: right ventricle, LV: left ventricle. (B) Quantization of cardiac ventricular area. *=*P* < 0.05, **=*P* < 0.01, *** =*P* < 0.001 compared with the age control group (C3 group); #=*P* < 0.05, ##=*P* < 0.01, ### =*P* < 0.001 compared with the indicated groups without exercise training.

We investigated the correlation between HW weight and tibia length. This value avoids external factors and more closely displays changes in HW weight for comparisons between groups. Comparisons of this ratio between each groups demonstrated that the ratio in experimental rats changed with HW weight. Table 1 indicates that the HW weight and heart chamber wall thickness (Fig. 2) increased proportionally with age. HW weight increased with age in the aging without exercise rats (A12, and A18). The HW weights of the exercise training groups (E3, AE12, and AE18) were reduced (*P* < 0.05). The exercise training effect was demonstrated in the young group (E3) and aging groups (AE12, AE18), and it exhibited a very significant difference.

### Histopathological tomography section analyses of cardiac tissue

Histopathological tomography analyses of whole hear tissues were performed using H&E staining (Fig. 2A). Images were viewed under a microscope and the control group C3 exhibited normal cardiac myocyte architecture and volume. However, after measured by imageJ (Fig. 2B), the heart sizes in aging rats (A12, and A18) were found to be increased, and heart walls were thickened. Heart chambers (left ventricular and right ventricle) narrowed with age, but this phenomenon was rescued in both aging with exercise training groups (AE12, and AE18).

### Exercise training prevented pathological hypertrophy in aged rat hearts

**Figure 3:**
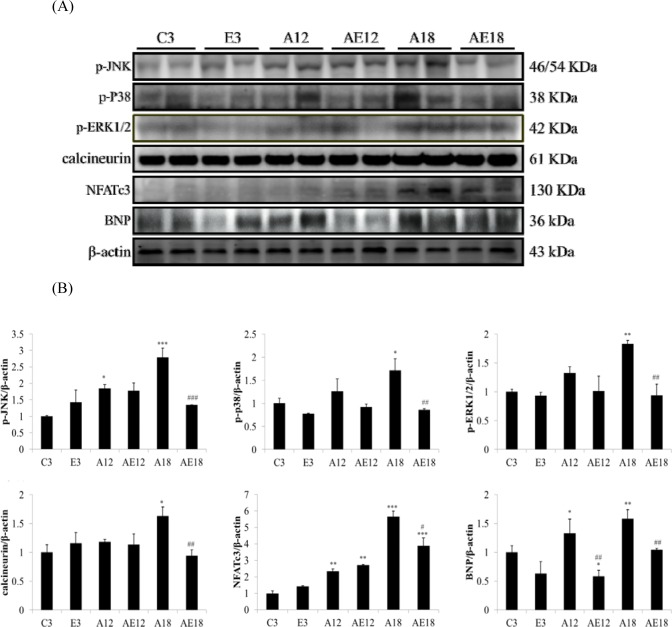
Representative hypertrophy protein products extracted from the left ventricles of two rats in each group: control rats (C3), aging rats (A12, A18), and aging, exercise-trained rats (E3, AE12, AE18) were measured using Western blotting β-actin was used as an internal control. *=*P* <0.05, **=*P* < 0.01, *** =*P* < 0.001 compared with the age control group (C3 group); #=*P* < 0.05, ##=*P* < 0.01, ### =*P* < 0.001 compared with the indicated groups without exercise training.

Our previous data demonstrated that aging induced heart weight gain (Table 1 and Fig. 2). We investigated whether aging affected the pathological hypertrophy pathway in cardiomyocytes. We determined the genes that are involved in pathological hypertrophy (Fig. 3A). The data demonstrated that upstream genes (p-JNK, p-p38 and p-ERK1/2) were up-regulated in aging groups (A12 and A18) compared with the C3 group, and the pathological hypertrophy transcription factors, NFATc3 and p-GATA4, were increased in aging rats hearts (A18). The cardiac hypertrophy marker B-type natriuretic peptide (BNP), which is up-regulated by NFATc3, was up-regulated compared to the control group. Aging with exercise training reversed the up-regulated expression of members of the pathological hypertrophy pathway. The protective effect in the A18 group was more significant (*P* < 0.01). These results indicated that exercise training reduced aging-induced cardiac pathological hypertrophy.

### Exercise training retarded cardiac fibrosis in aged rat hearts

**Figure 4:**
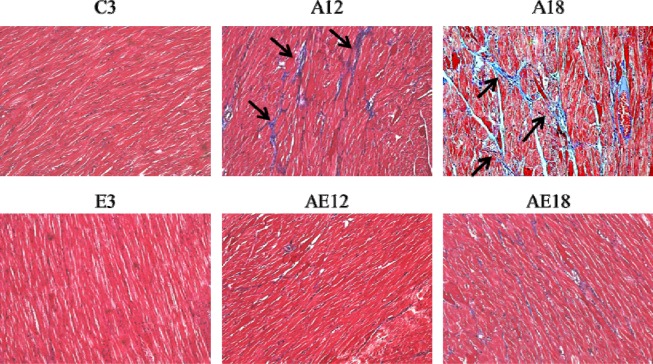
Masson's trichrome staining Cardiac tissue sections stained with Masson's trichrome staining buffer. Collagen accumulation is shown in blue color (black arrow). The myocardial architecture images were magnified ×400. The scale bar is 10 μm.

**Figure 5:**
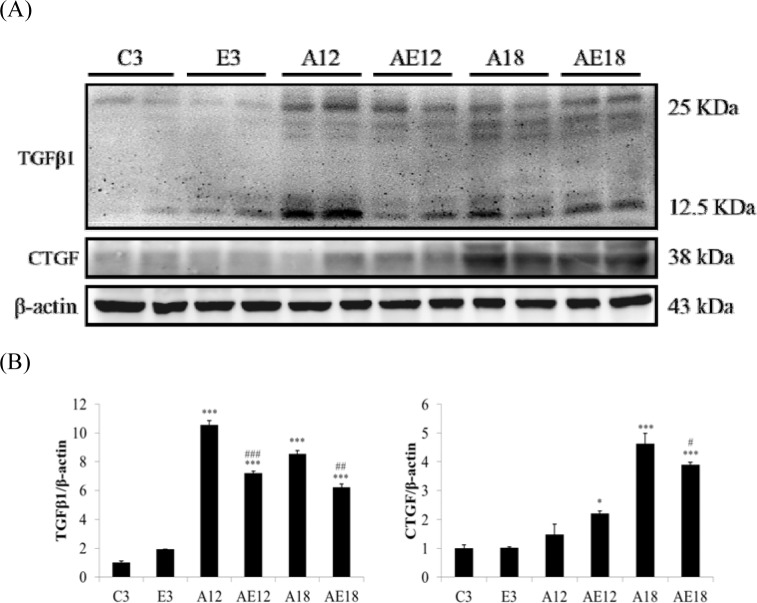
Representative protein products of the TGFβ-dependent fibrosis pathway extracted from the left ventricles of two rats in each group: control rats (C3), aging rats (A12, A18), and aging, exercise-trained rats (E3, AE12, AE18) were measured using Western blotting β-actin was used as an internal control. *=*P* < 0.05, **=*P* < 0.01, *** =*P* < 0.001 compared with the age control group (C3 group); #=*P* < 0.05, ##=*P* < 0.01, ### =*P*<0.001 compared with the indicated groups without exercise training.

**Figure 6:**
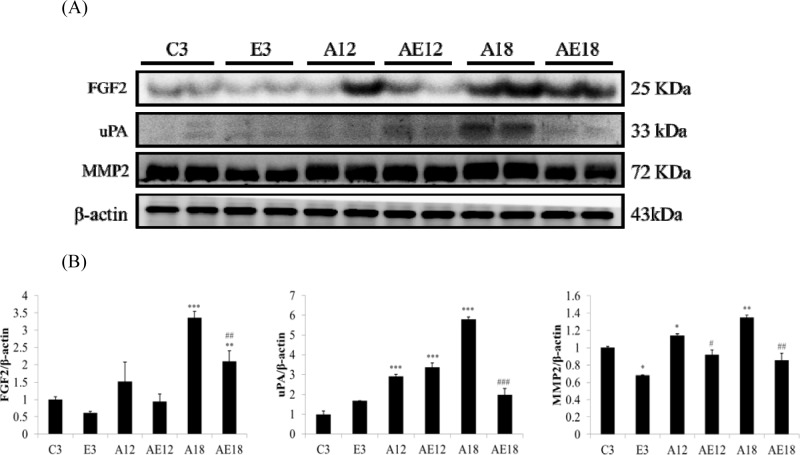
Representative protein products of the FGF2-dependent fibrosis pathway extracted from the left ventricles of two rats in each group: control rats (C3), aging rats (A12, A18), and aging, exercise-trained rats (E3, AE12, AE18) were measured using Western blotting β-actin was used as an internal control. *=*P*<0.05, **=*P* < 0.01, *** =*P* < 0.001 compared with the age control group (C3 group); #=*P* < 0.05, ##=*P*<0.01, ### =*P* < 0.001 compared with the indicated groups without exercise training.

Aging is a risk factor for heart failure. This study demonstrated that aging induced cardiac inflammation and pathological cardiac hypertrophy (Figs. 1-3), which follows the process of heart failure. We detected cardiac fibrosis using Masson's trichrome staining of heart tissue sections (Fig. 4). Collagen accumulation occurred with aging in the aging groups (A12 and A18). The collagen accumulation in the A18 group was more serious than the A12 and C3 groups. Exercise training reduced collagen accumulation in aging rats groups (AE12 and AE18). We also investigated the fibrosis molecular signaling pathways. Fig. 5A shows that TGFβ1, which is the ligand in the TGF-dependent fibrosis pathway, was up-regulated in the aging groups (A12 and A18) and resulted in CTGF overexpression compared to the control group (C3). The aging with exercise training groups (AE12 and AE18) exhibited a reversal of aging-activated TGF-dependent fibrosis pathway expression (Fig. 5A). We investigated FGF2/uPA/MMP2 in the cardiac fibrosis pathway. FGF2 expression was up-regulated in aged rats (A18), and aging also induced the expression of the down-stream genes uPA and MMP2 (Fig. 6). Notably, exercise training prevented aging-induced cardiac fibrosis in the AE18 group, after exercise training reduced FGF2, uPA and MMP2 expression.

## DISCUSSION

It is well known that reactive oxygen species (ROS) also play a role during aging, previous studies revealed that aging-associated cardiovascular disease involve the role of nicotinamide adenine dinucleotide phosphate-oxidase induced ROS accumulation resulting in cardiac inflammation and fibrosis [35-37]. Many researches have shown that drug or herbal extract mediated amelioration of cardiac injury is correlated with the extent of ROS scavenging [36, 38]. In this paper we revealed that moderate exercise can attenuate the aging-induced cardiac inflammation, hypertrophy and fibrosis injuries of rat hearts, however we need more experiments to verify whether correlation of ROS regulation and moderate exercise effect on cardiovascular disease in aged rats.

Our previous studies demonstrated that diabetes, high blood pressure, obesity and aging were CVD risk factors [39]. Apoptosis of cardiomyocytes always appeared in aging rat hearts [40]. Long-term inflammation is one of the major risk factors that leads to cardiac hypertrophy [41]. Our data demonstrated that cardiac inflammation existed in aging hearts, and this inflammation was induced by the activated NFκB signaling pathway. Figure 1 show that p-IKKα/β and p-Iκβ were up-regulated, which activated NFκB and up-regulated COX2 and iNOS expression. COX2 and iNOS are downstream genes of NFκB and key markers of inflammation. These data indicated aging resulted in cardiac inflammation, and exercise training prevented aging-induced cardiac inflammation, especially in elderly rats (A18 and AE18 groups) (Fig. 1). Other studies demonstrated an effect of exercise training on inflammation in adipose tissue, muscle and brain [42-44]. The present study demonstrated that the inflammatory pathway was up-regulated in aged rats hearts, and training with moderate exercise decreased inflammation in aging rats hearts (A18 and AE18) (Fig. 1).

Cardiac hypertrophy, or remodeling, includes changes in cell size and morphology [45]. Changes in pre-load, after-load or any pressure (*e.g.,* inflammation and hypertension) trigger cardiomyocyte remodeling, which involves hypertrophy and fibrosis. The overloading pressure promotes cardiomyocyte transformation from physiological hypertrophy to pathological hypertrophy, and pathological hypertrophy is an irreversible process in the heart [46]. No studies indicated whether moderate exercise training affected aging rat heart remodeling. Table 1, Figure 2 and Figure 4 show that aging induced cardiac remodeling. Whole heart weight and left ventricular wall thickness increased in the aging groups. Pathological hypertrophy can be divided into two types, concentric hypertrophy and eccentric hypertrophy. The heart wall thickens in concentric hypertrophy and the chamber size is reduced. The heart wall becomes thin or does not change in eccentric hypertrophy, and the chamber size is enlarged [47]. Our findings demonstrated that aging induced cardiac pathological concentric hypertrophy via ERK1/2/JNK and NFATc3 expression (Fig. 3). Notably, training with moderate exercise decreased the risk for cardiac hypertrophy via down-regulation of the concentric hypertrophy pathway.

We examined the mechanism of aging-induced cardiomyocyte apoptosis, which may occur through inflammation and cardiac hypertrophy, and whether exercise training rescued the injury. Aging induced collagen accumulation (Fig. 4) and cardiac fibrosis via the TGF-β and FGF2 pathways (Fig. 5 and Fig. 6). Notably, we clarified that aging-induced cardiac fibrosis primarily occurred through the FGF2/uPA/MMP2 pathway, and fibrosis in aged rats was more severe (Fig. 6). Exercise training decreased cardiac fibrosis in the aged rats group (AE18).

**Figure 7:**
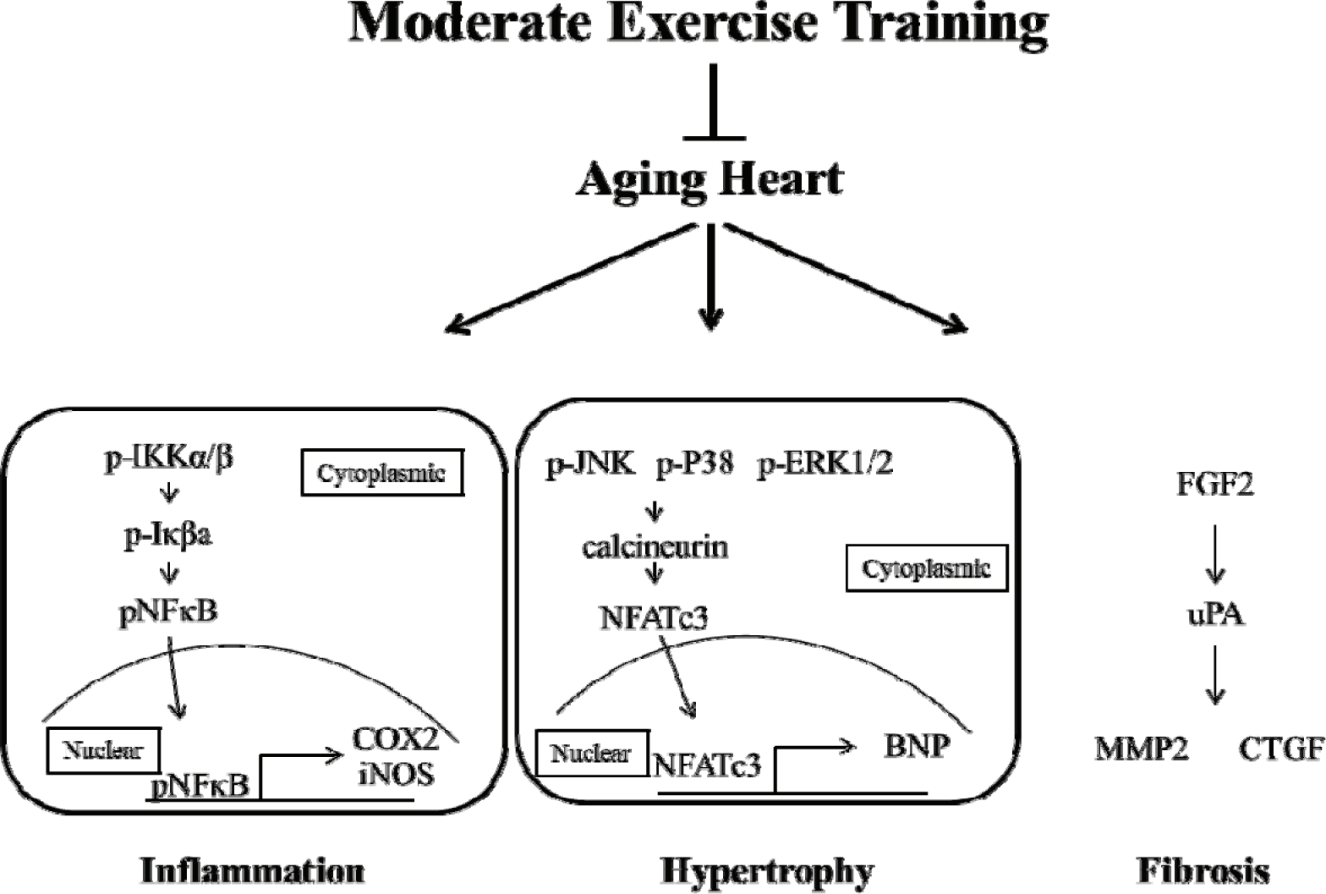
Moderate exercise training prevents aging-induced heart injury via down-regulated inflammation, hypertrophy and fibrosis

In conclusion, aging caused heart injury and activated the process of heart failure. Moderate exercise training decreased the heart injury via the down-regulation of inflammation, hypertrophy and fibrosis (Fig. 7). No significant injury in rat hearts was found in the young rats in the exercise training group (E3). These findings demonstrated that moderate exercise training provided cardio-protection in aged rat hearts via the down-regulation of cardiomyocyte inflammation and remodeling. Notably, exercise training is neither a treatment nor therapy, but it decreased aging injuries in rat hearts. These results demonstrate that moderate exercise training provide aging rat hearts a rest environment with increased cardiorespiratory function [48] and enhanced heart function and structure [49] to reduce the possibility of heart failure in aging hearts.

## MATERIALS AND METHODS

### Animal models

Twenty-six Sprague-Dawley (SD) male rats at different age stages were used. Ten 3-month-old and 12-month-old rats and six rats at 18 month olds were used. Each age group was randomly divided into two groups: C3: 3-month-old control group; E3: 3-month-old exercise training aging rats; A12: 12-month-old control group; AE12: 12-month-old exercise training aging rats; A18:18-month-old control group; and AE18: 18-month-old exercise training aging rats. No more than 3 rats were housed per cage. Room temperature was maintained at 22-24°C, and rats were housed on a 12-hour light-dark cycle. Rats were supplemented with standard laboratory chow (Lab Diet 5001; PMI Nutrition International Inc., Brentwood, MO, USA), and water was available ad libitum. The Institutional Animal Care and Use Committee of China Medical University, Taichung, Taiwan approved all protocols, and the principles of laboratory animal care (NIH publication) were followed.

### Exercise training protocol

The exercise training protocol was based on a previous protocol [7, 34]. Rats in the control group were soaked in the right amount of water, and the water temperature was 25±2°C. The depth of swimming pool (35 cm) could not lead to rats drowning, and there were only two rats swimming at the same time. Exercise training began for rats in the exercise group (E3, AE12, AE18) swam for 20 min per day for the first 2 weeks. Swimming training time was extended to 30 min per day at the third week. The swimming training time was extended to 1 h per day in the remaining weeks, and all of the swimming times were recorded 5 times weekly. Animals were sacrificed after the experimental period and heart tissues were collected. The left ventricle tissues were isolated, and the tibia length was measured.

### Tissue extraction

The entire heart weight was measured, and the left ventricle tissues from each group were isolated and washed 3 times in PBS buffer. Tissue samples (0.1 mg) were collected, and 1 mL lysis buffer (50 mM Tris-HCL, 1% NP-40, 0.5% Na-deoxycholate, 0.1% SDS, 150 mM NaCl, 2 mM EDTA, 50 mM NaF) was added to the mixture. The tissue was homogenized for 5 min and centrifuged at 2,200 rpm at 4°C. Samples were homogenized, centrifuged at 12,500 rpm, and a clean upper layer suspension was extracted. The centrifugation step was repeated, and a clean upper layer suspension was extracted.

### Heart slices area tomography analysis

The heart tissue cross-section were made from identical location and stained by hematoxylin and eosin. Heart chamber area was measured by imageJ software.

### Western blotting analysis

Tissue protein samples were mixed with 5× loading dye and placed on 95°C for 5 min. The samples were loaded onto sodium dodecyl sulfate polyacrylamide gel electrophoresis (SDS-PAGE). The upper SDS-PAGE was a 5 % gel, and the underlying gel was an 8, 10 or 12 % separating gel. The prepared SDS-gels were placed in a vertical electrophoresis system. The prepared protein samples were loaded into U-shaped wells. The gel electrophoresis system worked at 100 volts (V). The gel was removed after the electrophoresis was completed and covered with a polyvinylidene difluoride (PVDF) membrane. The gel and PVDF membrane were placed into a transfer tank at 90 V for 70 min at 4°C. The PVDF membrane was soaked in a 5 % blocking buffer/Tris-buffered saline (TBS)-fat-free milk solution on a shaker for 40 min at room temperature. The PVDF membrane was washed with TBS buffer 3 times for 10 min each and incubated with the primary antibody overnight at 4°C. The PVDF membrane was washed with TBS buffer 3 times for 10 min each and incubated with the secondary antibody for 1 hr at room temperature. Membrane data was collected using an LAS-4000 mini (GE Healthcare Life Sciences), and data were quantified using Image J software.

### Hematoxylin and eosin staining

Tissue sections were stained using hematoxylin and eosin (H&E). Sections were deparaffinized by immersion in xylene and stained with hematoxylin for 5 min. Sections were washed in double-distilled water (DDW) three times and placed in 85 % alcohol for 2 min. The sections were stained with eosin for 5 min and incubated in an ascending alcohol concentration gradient (70, 80, and 90 %) for 5 min. Sections were placed in 100 % alcohol for 5 min and twice in xylene for 1 min. Photomicrographs were detected using a microscope (OLYMPUS Microscope).

### Masson's trichrome staining

Rat heart tissue from each group was stored in 10% formalin for 2 weeks, dehydrated in an ascending series of alcohols (75%, 85%, 90%, and 100% alcohol, 5 min each) and embedded in paraffin wax. The 2 μm-thick paraffin sections were sliced from these paraffin-embedded tissue blocks. Tissue sections were de-paraffinized via immersion in xylene (3 times, 5 min each) and rehydrated using an descending series of alcohols (100%, 90%, 85%, and 75% alcohol, 5 min each). Biopsy samples were stained using Masson's trichrome stain to investigate heart morphological and fibrotic changes. Blue staining represented collagen accumulation. The results were visualized using an OLYMPUS microscope.

### Statistics

The experimental data are provided as the means ± SEM. Student ' s t test was used to compared two differences groups. One-way ANOVA was used for comparisons between multiple groups. P <0.05 was considered significant.
